# Conceptual Engineering Should be Empirical

**DOI:** 10.1007/s10670-025-00923-x

**Published:** 2025-02-11

**Authors:** Ethan Landes

**Affiliations:** University of Kent, Canterbury, United Kingdom

## Abstract

Conceptual engineering is a philosophical method that aims to design and spread conceptual and linguistic devices to cause meaningful changes in the world. So far, however, conceptual engineers have struggled to successfully spread the conceptual and linguistic entities they have designed to their target communities. This paper argues that conceptual engineering is far more likely to succeed if it incorporates empirical data and empirical methods. Because the causal factors influencing the successful propagation of linguistic or conceptual devices are as complicated and interwoven as they are, proper empirical research will greatly boost the likelihood that propagation is successful. In arguing for the superiority of empirical conceptual engineering over armchair-based conceptual engineering, this paper proposes a framework for understanding the causal forces at play in propagation. This is a three-part framework between the label of a lexical item, the psychological states associated with the lexical item, and the worldly things associated with the lexical item. By understanding the way causal forces affecting propagation play out at these three levels, conceptual engineers can better conceptualize, study, and harness the different causal forces affecting the success of their conceptual engineering projects.

## Introduction

Despite their apparent differences, conceptual engineering and experimental philosophy are natural bedfellows. Conceptual engineering is the ameliorative method of identifying deficiencies in our conceptual or linguistic repertoire, engineering a better replacement, and propagating said replacement (Isaac et al., 2022; Belleri, 2021; Chalmers, 2020). Experimental philosophy is the employment of empirical methods to answer traditional philosophical questions and is driven by the conviction that many traditional philosophical questions turn on complex and/or implicit empirical claims (Horvath & Koch, 2021; Machery, 2017). Despite their apparently different aims, both experimental philosophy and conceptual engineering represent alternatives to the methods of mainstream analytic philosophy (Torregrossa, 2024), and so unsurprisingly, there has been considerable work exploring the intersection of the two. We can split this work into three broad camps. Some have suggested conceptual engineering can or should be done empirically at some stage in the process, some have used extant empirical data to try to better understand conceptual engineering, and some have undertaken experimentally-informed conceptual engineering.

The largest set of papers examining the relationship between conceptual engineering and empirical methods have argued that conceptual engineering can or should be led by empirical methods. Nado (2019) argues a retooled experimental philosophy can aid functionalist accounts of conceptual engineering (see Nado, 2021) by helping discover the functions of various concepts, words, and so on. Focusing on aesthetics, Torregrossa (2024) argues experimental data can, and already has, revealed the defectiveness of aesthetic concepts, but Torregrossa remains sceptical that experimental philosophy can answer normative issues faced by conceptual engineers. In conversation with Torregrossa, Andow (2020) argues that normative truths are well within the grasp of experimental methods and maps out a framework for fully experimental conceptual engineering. In a different vein, Thomasson defends a conceptual engineering framework built around words as opposed to concepts or meanings in part because of the framework’s cohesion with empirical linguistics (2021, 11). In related discussions of Carnapian explication, others have defended both the role of experimental methods in identifying aspects of the explicandum (the precursor concept) (Koch, 2019; Schupbach, 2017; Shepherd & Justus, 2015), in testing how well the explicatum (the explicated concept) will spread (Pinder, 2017), and if an engineered concept succeeds at being an improvement over its precursor (Wakil, 2021).

Other papers have actively drawn from empirical methods to better understand conceptual engineering as a method. Fischer (2020) and Machery (2021) draw from their own past empirical work to argue that some conceptual changes will be more feasible than others. Drawing upon linguistics, Koslow (2022) argues that while meaning and concept change is chaotic when viewing change at the level of particular words, when examining language change at the macro level, predictable patterns emerge that we can study and harness to do conceptual engineering. Looking at the micro level, Landes (forthcoming) examines the propagation of "social distancing" and "coronavirus” early in the COVID-19 pandemic, arguing that the label "social distancing" potentially hindered propagation of the concept SOCIAL DISTANCING.

Third, some papers have actively incorporated empirical methods into conceptual engineering projects. In a series of papers, Machery and collaborators empirically explore the concept of INNATE, identify it as defective, and recommend that it be eliminated (Griffiths et al., 2009; Machery, 2017, 2021). They thus use the methods recommended by some of the authors above, using experimental findings of inconsistent judgements to motivate conceptual change. Napolitano and Reuter (2021) use experimental methods to demonstrate that folk find “conspiracy theory” to be negatively evaluative despite many philosophers’ insistence that the term is best analysed as neutral and descriptive. Napolitano and Reuter then argue for the introduction of "conspiratorial explanation" as a neutral counterpart to "conspiracy theory" (see also Reuter and Brun (2022) on “truth”). Landes and Reuter (preprint) look at conceptual revision, developing empirical methods to identify whether conceptual change has taken place and successfully revise default judgements participants made about PLANET to be in line with the IAU's, 2006 redefinition (IAU, 2006).

This paper belongs to the category of those who argue that conceptual engineering should involve empirical methods. Like Pinder (2017), I argue that conceptual engineers should use empirical approaches to improve the chances of success propagation (what Pinder calls *uptake*). Unlike Pinder, I set aside issues of propagation’s value, instead focusing on the nitty–gritty reality facing conceptual engineers who want to get their creations out into the world. My central argument is therefore the following: Regardless of the conceptual engineering framework. if conceptual engineers care about propagation, they should deploy empirical methods. If they do not, propagation is *far* less likely to succeed. This is because the sorts of causal factors that affect the success or failure of propagation are complicated and difficult to anticipate but within the purview of the empirical methods of fields like psychology, sociology, and linguistics.

Three clarifications about this argument are in order. First, this argument is instrumental (see also Wakil, 2021). Empirical work is not necessary for successful propagation, as revealed by astronomers’ successful redefinition of “planet”. However, for every “planet”, there are countless candidates for revision currently mouldering in obscurity in academic journals. If we want our project to avoid this fate, we can greatly increase our chances by getting our hands dirty with empirical research. Second, this paper is meant to be framework-neutral. The conclusion does not depend on the specifics of any given conceptual engineering framework, and the paper’s discussion of causal forces is as neutral about the nature of conceptual engineering as possible. Third, the argument proceeds by exploring *some* of the causal forces affecting propagation to demonstrate that ignorance of these forces will harm propagation and that empirical knowledge about them will help propagation. To frame discussion of these forces, I develop a meta-framework for conceptualizing the causal forces relevant to propagation. This framework is centred around a single lexical item such as a word or phrase, and distinguishes its form, its associated mental states, and its associated extra-mental entities. This framing is intended to help conceptual engineers conceptualize, study, and harness the causal factors affecting propagation, but in relationship to the paper’s thesis, the mapping is merely a device to frame the paper’s discussion of just how complicated and unpredictable propagation can be—at least when one is confined to the armchair.

Section 2 introduces the tripartite meta-framework, and Sects. 3 to 6 each explore a different type of interaction on the meta-framework. The end of each section explores why ignorance of the relevant causal relations hurts propagation, why empirical methods would improve our odds, and indicates which empirical methods would help. Section 7 explores the consequences of the previous sections. Section 7.1 uses semantic externalist conceptual engineering to illustrate what an empirically informed propagation effort should consider in light of the paper’s argument. Section 7.2 argues that the complexity of causal forces means that conceptual engineers need to look beyond engineering individual words, meanings, or concepts and instead engineer *packages* of labels, mental states, and extra-mental changes.

## Labels, Thoughts, and the World

No single paper can do justice to the complex entangled web of causal connections relevant to conceptual engineering. Instead, the goal here is to develop a theoretically neutral meta-framework for thinking about that web to highlight and discuss the complexity of empirical questions facing conceptual engineers during propagation. This section sets up the rest of the paper while offering a useful metaphysics for engaging in conceptual engineering, regardless of how conceptual engineering is construed.^1^ This metaphysics splits reality into three parts based on its relationship to lexical items, namely, the label the lexical item uses as a vehicle, what psychological states a language user has associated with the lexical item, and what stuff in the world is associated with the lexical item.

The *label* is a lexical item’s appearance—the connections of letters and sounds that constitute the vehicle by which a concept or meaning is expressed by a language user. The label is what a lexical item looks and sounds like, which, as will be discussed below, can carry a sizable amount of information. Saussure has a similar notion of the *signifier*, the vehicle by which concepts (*the signified*) are communicated. In contrast to Saussure, I will talk about labels as physical manifestations of language whereas signifiers are psychological representations of such manifestations (2011, 66).

*Thought* will refer to the entities grounded in our token brain states or mental states. That is, thought is the stuff in our head associated with a lexical item.^2^ Since the notion is meant to be theory-neutral, how the category of thought is populated will depend on a whole host of theoretical commitments and will differ from reader to reader and conceptual engineering framework to conceptual engineering framework. For example, content internalists will put conceptual content in the category of thought (Machery, 2017; Pollock, 2021), whereas content externalists (Sawyer, 2020a; Scharp, 2013) or concept eliminativists (Cappelen, 2018; Machery, 2009) will not. The same is also true of internalists, externalists, and eliminativists about semantic content. What is not up for philosophical debate, however, is that our mind is structured in ways that are (fairly) stable and (partially) individualistic. The exact details of this structure, however, are still being uncovered by neuroscience, cognitive science, philosophy of mind, and other disciplines.

The third category, the *world*, is a catch-all to cover everything external to us, including both physical things and abstracta. Like before, which abstracta exist in the world depends on a whole host of philosophical commitments, and “world” in this context is meant to be a neutral term that can be applied to any set of metaphysical commitments that admits to the existence of a world beyond our minds.

Labels, thoughts, and the world, as understood and discussed here, will be talked about as being unified around a single lexical item. Take “dog”. The *label* “dog” has three letters. At the level of *thought*, we all have associations with “dog”. In my case, “dog” evokes images of my puppy, a dachshund named Fergus. In the *world*, “dog” is associated with Fergus himself, other dogs, and our social norms about dogs. Depending on your theoretical commitments, the worldly entities associated with “dog” may include the natural kind Dog, the semantic value of “dog”, the Fregean concept DOG, the causal-historical chain of “dog”, and other abstracta.

These three categories are useful to conceptual engineers during propagation because they capture two related types of joints relevant to propagation’s success. First, they correspond to different aspects of propagation. Most conceptual engineers want to fix something beyond just our beliefs and other token representations; they want to fix patterns of injustice, improve inquiry, guide technological innovation, and make other changes to the world (Isaac et al., 2022, 4–5). To bring about such change, a hearts and minds campaign is inevitable, as the right people need to be brought on board to bring about the larger worldly change (more on this in Sects. 5–6). That is, phenomena at the level of thought must be targeted, even if just as an intermediary step to something bigger. Serving as a vehicle for all of this, the label acts as the tangible linguistic foci of propagation.

The three categories also pick out the key causal joints of propagation. Tinkering with labels, thoughts, or the world are fundamentally different causal projects and require different approaches. A campaign to change the colour of maple tree leaves requires very different actions than a campaign to change people’s beliefs about the colour of maple tree leaves, although the success of one may affect the success of the other. Similar differences face conceptual engineering. A campaign to change the inferences language users should make in response to a statement-type (a worldly change) requires very different actions than a campaign to change the inferences a single listener in fact makes in response to a token statement (a change of thought). The label, while in a sense a part of the world, plays a unique causal role during propagation due to its salience during language use (as we will see in Sect. 3) and is largely under the control of the conceptual engineer (subject to some limits discussed in Sect. 4). Therefore, the causal factors relevant to labels are best discussed separately from causal factors relevant to the world more broadly.

These causal joints are additionally important because labels, thoughts, and the world are causally connected in ways that affect propagation’s success. While it might seem strange to say the label “dog” is causally connected to the (externalist) semantic value of “dog”, they indirectly are. “Dog” is causally related to our cognitive states associated with dogs and such cognitive states are causally connected to the world, including the parts of the world that ground externalist semantic values.^3^ There are four directions of causation relevant to conceptual engineering—label to thought, thought to label, thought to world, and world to thought—and each will be looked at in turn in the coming sections. Readers are invited to reflect on what they take to be the targets of conceptual engineering (whether words, generics, concept-tokens, concept-types, meaning-tokens, meaning-types, etc.), consider where these targets sit in the meta-framework, and consider how this affects the empirical considerations and methods highlighted below.

## Label to Thought

We are used to thinking about the causal connection between language and thought in relation to expression and interpretation. We hear a sentence and in response experience certain mental imagery or form certain beliefs. However, the causal relationship between words and thought is far more complex than just sentential interpretation, as what a label looks and sounds like influences our linguistic and extralinguistic representations. This is particularly clear in capitalized descriptions, such as “the Renaissance”, “the Giant’s Causeway”, and “the Rocky Mountains”, which straddle the line between definite descriptions and proper names (Rabern, 2015). To see the epistemically-available information that can be carried by the form of capitalized descriptions and how such information is different from other types of non-semantic information carried by language, imagine that we are almost entirely ignorant of European history and a friend says to us “My grandfather fought in the Spanish Civil War.” Even if we have never heard of the Spanish Civil War nor encountered the proper name “the Spanish Civil War”, from that sentence we can nonetheless reasonably infer things both about the referent of the proper name and Spanish history more generally.

First, we can use syntactic information to infer things about the name’s referent. Indeed, children as young as two years old use syntactic information and the meanings of prepositions to infer semantic information (Lidz et al., 2017; St. Pierre and Johnson, 2021). The preposition “in” tells us the referent of the “Spanish Civil War” is an event the grandfather took part in as opposed to an opponent he fought (compare to: “my grandfather *fought* the Spanish Civil War”). Second, we can use contextual information to infer things about the name’s referent. Given grandfathers are typically 40 to 80 years older than their grandchildren, we can reasonably infer a general timeframe of the event from the age of the speaker.

Setting these two inferences aside, there is still non-semantic information that we can reasonably infer from the sentence “My grandfather fought in the Spanish Civil War”. Namely, we can infer that Spain had a civil war. This might look like an inference based on the term’s semantics or syntax, but capitalized descriptions do not gain their semantics through the composition of their parts (Rabern, 2015). If Spain had a second civil war, “the Spanish civil war” would be an empty definite description, but “the Spanish Civil War” could remain the name of the conflict in the 1930s. Therefore, despite having a syntax that resembles definite descriptions, capitalized descriptions have semantics that work like proper names in that they are rigid and non-compositional (Rabern, 2015).

This introduces a puzzle. How can we infer that Spain had a civil war from the term “the Spanish Civil War” if the meaning of “the Spanish Civil War” is non-compositional? Simply put, words often wear their meaning on their sleeves. Even though the relationship between a lexical item’s form and its meaning and reference is arbitrary in theory, it is not arbitrary in practice (Grote and Linz, 2008; Dingemanse et al., 2015; Winter et al., 2017). Across our lexicon, there are regularities in the relationship between lexical items’ labels and their semantic properties. These regularities can in turn justify language users’ semantic and object-level beliefs.

Multiple notions exist in semiotics and linguistics to describe such regularities. *Transparency* is the degree to which a language user can infer the meaning of a multimorphemic word from the meaning of its parts (see Libben, 1998). This is a property of compound nouns, terms with affixes, and other multi-part labels relative to both individuals and meanings. For example, “reader” (a person who reads) is for most English speakers a transparent combination of the root *read* and suffix *-er*, whereas “reader” (the rank below professor in the UK) is for most *opaque*, i.e., not transparent. *Iconicity* is the resemblance between the form of a linguistic sign and its meaning. Sometimes iconicity is straightforward—the American Sign Language sign for *to drink* looks like someone is taking a drink from a glass with their right hand (see Grote and Linz, 2008; Baus et al., 2013). *Ideophones* are words that resemble sensory experiences related to the meaning (Dingemanse, 2012, 2018). These include *onomatopoeias*, such as “whoosh”, “whack”, and “moo” and iconic *phonesthemes*, or systematic associations between certain sounds and meanings, such as the association of “cr-” in English with abrupt sounds—e.g., “crack”, “crunch”, “creak”, and “crash” (Mompean et al., 2020). *Systematicity* is the statistical regularity between form and meaning. In contrast to iconicity and transparency, systematicity is often unprincipled.For example, compared with English verbs, on average English nouns have more syllables, are more likely to start with bilabials (e.g., b, m, p), and have more vowels as a percentage of total word sounds (Cassidy & Kelly, 2001; Monaghan et al., 2007).

Due to such regularities between form and meaning, our ability to infer semantic and object-level information from labels extends beyond capitalized descriptions like “the Rocky Mountains.” To illustrate, consider five object-level inferences that would be supported by the information conveyed by five different labels if the listener was unfamiliar with those labels:

- **Proper name**: “I visited *Rožďalovice.*”
  o *Conveyed information:* The speaker was in a historically Slavic part of Europe.
- **Multimorphemic adjective**: “The house was *decagonal*”
  o *Conveyed information*: The house had 10 sides.
- **Compound noun**: “I saw a *bluebird.*”
  o *Conveyed information*: The speaker saw a blue-coloured bird.
- **Slightly opaque noun phrase**: “She cooked with *clarified butter.*”^4^
  o *Conveyed information*: The butter had been cleared up in some way.
- **Monomorphemic word**: “There was a *whack.*”
  o *Conveyed information*: There was a sound that ended sharply.

To talk about the ways labels carry information, call the property on display in the examples above a label’s *informativeness*. A lexical item is informative when its form carries information that a language user is apt to use to infer useful or true information, such as about the lexical item’s meaning or referent.^5^ In contrast to informativeness, the information language users are apt to infer from a label can also be harmful or false. For example, here are five *misleading* labels that might cause false beliefs about the world:

- **Proper name**: “I visited *Trumpington.*”
  o *Misleading information*: The location is not related to the former US president.
- **Multimorphemic adjective**: “The gas is *inflammable*.”
  o *Misleading information*: The gas is flammable, not nonflammable (Koslow, 2022, 16).
- **Compound noun**: “I ate a *blackberry.*”
  o *Misleading information*: Blackberries are not technically berries.
- **Slightly opaque noun phrase**: “Please *social distance*.”
  o *Misleading information*: The practice primarily involves physical or epidemiological distancing with other people, not distancing that is *social* (see Landes forthcoming).
- **Monomorphemic word**: “Pigs *oink*.*”*
  o *Misleading information*: The grumbling noises pigs make sound nothing like “oink”.

In the cases of informative or misleading language, the label itself enables inferences, which then impacts the mental representations we have, not just about the language, but about other things as well. In this way, labels causally influence our beliefs, mental imagery, and other mental states. This is not to say such information or inferences ground, constitute, or give rise to the lexical item’s meaning or referent (although they might on some metasemantic accounts). Rather, these labels influence people’s linguistic and non-linguistic beliefs and other token psychological states related to the lexical item.

Informativeness and misleadingness are a three-way relationship between labels, thoughts, and the world that describe both the extent the appearance of a lexical item is prone to cause certain mental states and how those mental states line up with the world. An informative label will tend to cause mental states that are true, fitting, apt, etc., whereas a misleading label will tend to cause mental states that are false, unfitting, defective, etc., Importantly, while the relationship between label and thought is causal, the relationship between thought and the world is not. Moreover, the relationship between thought and world depends on the framework. Functional or pragmatic frameworks (Isaac, 2021; Nado, 2021; Thomasson, 2020) may frame informativeness and misleadingness in terms of usefulness or efficacy, whereas realist frameworks (Cappelen, 2018; Sawyer, 2020b; Scharp, 2013) may frame them in terms of truth or defectiveness.

### Why Empirical Research Matters

Harnessing label-to-thought causal forces will greatly improve the odds of successful propagation. Label-to-thought causal forces ultimately create or reduce the friction of propagation by influencing what inferences people make about novel or revised linguistic devices. Fighting unwanted influences caused by a misleading label will increase the required effort to propagate a desired change, while an informative label will do the opposite. Misleading labels will hurt propagation by, for example, confusing people about a novel term’s meaning (see Landes forthcoming). Informative labels will instead speed up learning by combining known ideas in novel and illuminating ways—as was the case with “sexual harassment” (Maitra, 2018) and “mansplaining” (Koslow, 2022) —helping spread concepts and beliefs.

The epistemic problem conceptual engineers face is that what information is drawn from a label will differ from person to person, and what is informative to one person might be misleading to most other people. Conceptual engineers are not an accurate cross-section of language users, and what will be informative or misleading to us may not be what is informative or misleading to everyone else. Consequently, conceptual engineers will be more likely to pick an informative label if they check with the community in which they aim to propagate a cognitive or linguistic item. This empirical work could be quantitative, such as studies on how labels affect semantic and non-semantic judgments (see Majnemer & Meibauer, 2023; Mandelbaum et al., 2024), or it could be qualitative work such as focus groups or interviews (Sørensen et al., 2021). Therefore, empirically-informed projects will be in better a position to choose labels that improve the chance of successful propagation.

## Thought to Label

Informativeness and misleadingness are part of a larger feedback loop that exists between a lexical item’s form and the mental representations associated with it. While, as discussed in the previous section, the information carried by a term can cause beliefs and associations, our beliefs, associations, and other parts of our psychology influence what labels appear in the lexicon. Notice I am not merely saying that our psychology influences what vocabulary we pick up from other people. While this is true, the point is subtler: our psychology influences what form individual entries in our vocabulary—and the wider lexicon—take.

To discuss this, start with two properties of the relationship between a term’s form and our mental representations, a term’s fit and stickiness. *Fit* is the felt aptness of a label’s form, whereas *stickiness* is the memorability of a label-meaning or label-concept pair. A term’s fit and stickiness are related to informativeness, since an informative term will often feel apt and prove memorable. “The Spanish Civil War” is both a fitting and memorable name for Spain’s civil war. Nonetheless, fit and stickiness are both causally and conceptually distinct from informativeness. Informativeness without fit occurs when a label conveys information in a way that seems inappropriate, such as calling the Judeo-Christian God “Super-duper-extra-ultimately-strong-smart-and-good-thing”. Informativeness without stickiness occurs when a label carries information but is difficult to remember, such as calling the Spanish Civil War, “the 1936 to 1939 War Between Spanish Republicans, Syndicalists, and Communists vs Spanish Nationalists, Falangists, and Monarchists”. If someone earnestly tried to introduce this as the name for the war, people would either forget it or forgo it for something easier and more memorable—like “the Spanish Civil War”. Terms do not need to be informative to fit or to be sticky, though. Misleading terms are often socially problematic exactly because they are sticky, and brands often have meaningless but memorable names.

Fit and stickiness are ways our psychology puts causal pressure on what labels are in the lexicon. Fit puts causal pressure on what labels are introduced in the first place. We intuitively understand that some words are better labels for a thing than others and will often hunt around for the right label for a new idea. In contrast, stickiness puts causal pressure on what labels remain in the lexicon once they are introduced (Monaghan et al., 2007). Terms that are not sticky can be introduced, but they will not be readily retained or used by language users. Consequently, they may not enter the lexicon or may die out between generations of language users.

Fit and stickiness are illustrations of the larger network of causal pressures our beliefs, desires, associations, etc., put on a lexicon. In drawing upon empirical pragmatics and diachronic semantics to aid in the understanding of conceptual engineering, Koslow (2022) catalogues several phenomena that are thought-to-label causal forces. *Homonymy avoidance* is people’s tendency to avoid and find ways around confusing ambiguities (Koslow, 2022, 94–96). When a label is problematically homonymous or polysemous—there are often no salient contextual or syntactic clues to disambiguate between senses—speakers will avoid the label to avoid confusion. Like fit, this will prevent the introduction of certain word-meaning pairs, and like stickiness, this will cause people to avoid the label. *Convenience* and *efficiency* are how easy a speech act containing the label is to make and the ratio of energy to communicated information, respectively (Koslow, 2022, 94). As demonstrated above, informative labels are often very inconvenient and inefficient at referring to the referent. Convenience and fit come apart when a label seems fitting but is difficult to pronounce or spell, and efficiency and fit come apart when a label is short but does not seem fitting (e.g., calling the Spanish Civil War “Ba”). Convenience and efficiency put pressure on which labels are introduced and which are propagated, as the people who coin language will often strive for snappy language and language users will, all things being equal, prefer easier ways of communicating.

### Why Empirical Research Matters

Like label-to-thought causation, thought-to-label causation determines how much friction a propagation effort faces and, consequently, its odds of success. Fundamentally, thought-to-label causal forces affect a word-meaning or word-concept pair's staying power. Concepts or meanings that are paired with labels that feel right or labels that have some other sort of aesthetic draw will spread more readily than concepts or meanings paired with awkward labels. We cannot accurately predict from the armchair how a target community views the aesthetic properties of such a pair. We may be very different demographically from our target community, we may be too close to our creation to realize what it looks like for the first time, or we may just have idiosyncratic judgements. The community’s interpretation and aesthetic preferences can be revealed by, for example, having a focus group comment on how they like or dislike certain label-meaning pairs, much like market researchers test brand names. To test stickiness, conceptual engineers could measure how well participants remember word-concept pairs over time through multiple-choice vocabulary tests, open-ended redefinition tasks, or text comprehension tasks. Such studies would enable conceptual engineers to choose more memorable and attractive label-meaning or label-concept pairs, increasing the chances that members of the target community will remember, adopt, and spread the product of conceptual engineering.

## Thought to World

Now that the two causal directions between labels and thought have been mapped, it is time to turn to the two causal directions between thought and the world. In its most familiar forms, the causal relationship from thought to world is mundane. This morning, I was hungry, so I put cereal and milk together in a bowl. My thoughts influenced the world by reducing the amount of cereal in my cupboard, and the world influenced my thoughts, as the amount of cereal led to my belief I did not have to run to the grocery store.

Conceptual engineers are interested in far larger and more interesting changes in the world than how my beliefs and desires influence the contents of my cupboard. Many conceptual engineers are interested in changing social norms as a means to some end, such as changing meaning (Nimtz, 2021; Thomasson, 2021) or ending oppressive power structures (Haslanger, 2000; Manne, 2018). Because social norms and institutions are grounded in the beliefs, habits, and expectations of individuals, what social norms and institutions exist depend in large part on what we think they are and should be. This includes formal power structures such as law. The legal institution of marriage expanded to include same-sex couples because enough people situated in the right place in society believed the institution should change and were motivated to change it. This interplay between institutions, norms, and scientific facts on one side, and beliefs, desires, and motivations on the other, are the most relevant causal relationships between thought and the world for conceptual engineers.

To clarify discussion, I will follow Isaac et al. (2022) in distinguishing between the purposes, goals, and targets of conceptual engineering. The *purpose* of conceptual engineering is the final aim of the conceptual engineering project. These are generally things in the world, such as power structures, behaviours, truth-values, and kindhood, whose change would achieve some desired good. What makes conceptual engineering unique compared to other activist activities is that conceptual engineering aims to achieve its purpose through some linguistic or conceptual change. These linguistic or conceptual changes are the *goal* of conceptual engineering, and the linguistic or conceptual entities they hope to change are the *target*. It is because of the interplay between a project’s target, purpose, and goal that conceptual engineers need to pay attention to the causal forces between thought and the world. Thought-to-world causation in particular affects whether the necessary changes (that is, the project’s goal) are propagated in the right way to fulfil the project’s purpose.

First consider frameworks whose target is at the level of thought, such as individually-grounded understandings of speaker meaning, conceptual content, or meaning (e.g., Machery, 2017; Pinder, 2021; Plunkett & Sundell, 2013). Even though the target of conceptual engineering for these frameworks is at the level of thought, thought-to-world causation still matters to the success of a conceptual engineering project. The targets are means to an end, and so the targets are only worth engaging in if targeted mental representations (speaker meaning, conceptual content, meaning, etc.) have the desired effect. This cannot happen if changing the target backfires or proves to be epiphenomenal. Therefore, conceptual engineers with targets at the level of thought need to study thought-to-world causation to understand whether people will react to the propagated target in a way that brings about the project’s ultimate purpose.

Now consider frameworks with targets at the level of the world—such as norms, concepts, or legal institutions, (Cappelen, 2018; Haslanger, 2000; Scharp, 2013). Such frameworks need to be aware of how their attempts at revision or replacement will be interpreted. While changes in thought are not the target or goal of such projects, changes in thought in response to intentional propagation will affect the grounds of their target. While concepts (understood here as mind-independent entities), semantic values (similarly understood), or linguistic practices are not thought-level entities, their grounds often are. Such grounds include collective linguistic beliefs, collective linguistic use (Sawyer, 2020a), or collective intentions to take part in some reference chain (Sterken, 2020; Riggs, 2019, 11). The individual instantiations of these grounds—namely linguistic beliefs, linguistic habits of use, and linguistic intentions—are token mental states. The same holds for other worldly changes, such as norms and institutions. Norms and institutions exist because they are instantiated in the minds of the people taking part in them, often in extraordinarily complex ways. Therefore, changes in the world will generally require changes in token mental states, and world-level engineers cannot escape the need for research into how thought and the world interact.

### Why Empirical Research Matters

Stepping back, there are two broad families of thought-to-world questions facing conceptual engineers, and empirical study of both will prevent conceptual engineers from wasting their time pursuing pointless or even harmful conceptual or linguistic changes. First, how are the entities conceptual engineers hope to change structured? Here we want to understand how individuals and their mental states contribute to the structures that conceptual engineers hope to alter as part of their project’s purpose.^6^ Second, how will specific interventions change the behaviour, grounds, or norms underlying such entities? We need to understand whether our attempts to spread our revision will lead to the desired ends. Conceptual engineers’ ability to answer both sorts of questions will affect the efficacy of their propagation efforts, as it will determine whether the right messages are spread to the right people to fulfil the project’s purpose.

It would be hubris to answer both questions without relying on empirical work. From the armchair, we can guess what the right messages are and who the right people are. However, our understanding would be limited to our own experience and personal understanding of the often-opaque factors that, in the first question, constitute the ways we contribute to larger social structures, and in the second question, have changed the ways we contribute to larger social structures. Conceptual engineers would be much more likely to succeed at their stated aims if they drew from fields that study social structures empirically, such as anthropology and sociology. Methods like social network analysis would allow conceptual engineers to understand how ideas flow throughout a population and better deploy what resources have been allotted to propagation, and ethnography could reveal how members of relevant institutions react to change and external influences (see also Pinder, 2017, 457–59). Going in blind might still result in successful propagation, but the more a conceptual engineer knows about thought-to-world forces, the more confident they can be that they are not wasting their time on projects that will not pay off.

## World to Thought

The most relevant world-to-thought causal relations for conceptual engineering are those that affect the motivation of the engineer and the target audience of an engineering project. We have the conceptual or linguistic problems we have because there is some friction between our representational devices, desires, and the world. Accordingly, the perceived state of the world will influence what projects are seen as worth doing and worth buying into.

Looking first at the engineer, contingent facts about the world affect the desiderata and/or salience of desiderata for conceptual engineering. Consider the redefinition of “planet” (IAU, 2006). In 2005 astronomers discovered Eris, an object with a similar size and orbit to Pluto. This confirmed astronomers’ growing suspicion that the orbit beyond Neptune had dozens or even hundreds of Pluto-sized objects, and ultimately led to the redefinition of “planet” (Brown, 2006; Chang, 2022). Even if we grant the IAU’s definition correctly captures the joints of planethood, the revision was driven by the discovery of Eris. If the solar system had formed differently and Pluto was the only sizable object beyond Neptune, then the necessary motivation to engineer “planet” would not have arisen at the time it did in the community it did with the urgency it did. Friction between the world and astronomers’ mental states caused the revision.

In addition to the motivations of engineers, world-to-thought causal forces also affect the motivations of the subjects of propagation efforts.^7^ For an engineered linguistic or cognitive entity to spread, people need to buy into the change and spread it (whether consciously or unconsciously). We know that “technically” tomatoes are fruit and eggplants are berries, but we still use the words in a non-technical way because the costs of changing how we speak and think do not outweigh the benefits of lining up with experts’ technical usage (Abbott, 1997). After all, very few of us want eggplant in our berry parfaits. If non-technical uses of “berry” suddenly led to problems while ordering food, many of us would quickly change our tune.

### Why Empirical Research Matters

Conceptual engineers should study how the world affects people’s motivations. Like the other causal interactions discussed here, if the wrong forces are in place—if worldly facts do not motivate a project—propagation will face significant and possibly fatal headwinds. Changes that are instead seen as trendy, necessary, or useful will take significantly less work to propagate because people will be motivated to adopt and spread the changes. Some of this will be outside of the conceptual engineers’ control, as we cannot control what astronomers find in the icy reaches of the Kuiper belt. Nonetheless, many things are in our control, as marketing campaigns are a world-to-thought mechanism that use world-based stimuli to change perceptions of products. Notably, good marketing campaigns are often the product of careful consumer testing and research.

Therefore, conceptual engineering will be more likely to achieve their projects’ purpose if they know what relevant worldly conditions currently exist, how such conditions are perceived, and what world-based messaging will motivate people to adopt a cognitive or linguistic entity. Not to belabour the point, but from the armchair, we can only speculate about perceptions outside of our academic bubble. Surveying a representative cross-section of Americans about their deferential attitudes towards astronomers and investigating how likely it is that a press release by the International Astronomers’ Union will gain media attention will tell us way more about whether a redefinition of “planet” by the IAU is likely to motivate non-astronomers to change their use of “planet” than armchair speculation will. If conceptual engineers do not examine the relevant empirical data—whether via sociology, psychology, market research, astronomy, or other empirical methods—conceptual engineers face much higher risks of all the work they put into designing conceptual or linguistic devices turning into Quixotic projects that no one else ever cares about.

## From Label to World and Back Again

In mapping some of the causal forces that factor into successful propagation, I have argued that the causal complexity of propagation means empirical research will make successful propagation far more likely. This section explores what this means in practice. Section 7.1 uses semantic externalist conceptual engineering to illustrate that even conceptual engineering projects with abstract targets gain by engaging in empirical research. This is because while the targets of conceptual engineering are often abstract, the grounds of such targets are often not. Section 7.2 argues the lessons here necessitate a holistic approach to conceptual engineering. Conceptual engineers should not focus on a single device to engineer and instead should focus on engineering broader clusters of causally interwoven labels, thoughts, and worldly changes.

### An Illustration

Imagine we as conceptual engineers understand the target of conceptual engineering to be the externalist semantic values of words because we want to change the truth conditions of sentences for epistemic reasons (see Isaac et al., 2022, 15). Much has been written about what sort of metasemantic moves and changes need to happen to revise meaning on an externalist picture (Ball, 2020; Cappelen, 2018; Koch, 2021; Pollock, 2019; Sterken, 2020). What is true across the board, however, is that for a meaning to exist on externalist accounts, the word needs to first be baptized or anchored in some way and then the word-meaning pair needs to be propagated throughout the linguistic community (Sterken, 2020). There are significant differences in what this propagation requires or consists of, and depending on the account it might be that experts must change their minds, causal chains must break, linguistic norms must change, or patterns of use must change.

In each of these cases, changing semantic meaning requires changing thought in some way. Collective linguistic intentions, norms, patterns of use, and other grounds of meaning ultimately emerge because of the beliefs, desires, and other mental states of individuals. Causal chains are perpetuated because people know about the causal chains and are motivated to use them. Someone who has never encountered the causal chain associated with “Caesar” cannot speak with the intention to take part in it, nor will they want to if they think Caesar was just some guy from Rome. Similarly, with linguistic norms, someone cannot take part in a norm they have not encountered, and if they dislike the norm, they will avoid participating in it (see Thomasson, 2021; Nimtz, 2021).

We can, as conceptual engineers, coin, baptize, or anchor as many new meanings for words as we want. However, it is a necessary (but not sufficient) condition for meaning change that people adopt our lexical innovations. Therefore, we must think about how and why people might pick up or resist the new meaning. Accordingly, successful propagation requires harnessing label-to-thought causation to make sure the label is snappy and not misleading as well as world-to-thought causation to make sure people are motivated to change their ways. On a Kripkean causal-historical story (Kripke, 1980; Soames, 2003), this will involve figuring out what will make people adopt a causal chain going back to the baptism. On a Burge-style view where meaning is determined by experts (Ball, 2020; Burge, 1979), changing meaning will involve identifying experts and then identifying and targeting what will change their behaviour as a group. Both accounts will be aided by understanding various empirical considerations in sociology, psychology, cognitive science, historical linguistics, and marketing.

The web of causal influences on propagation is far more complicated than presented here. This paper has only described some of the causal forces that would affect the success of propagation and some of the ways the relevant causal forces interact. Nonetheless discussion here is meant to illustrate how engineering even something as abstract as externalist semantic value cannot solely focus on engineering abstracta. To successfully propagate a meaning, we need to pay attention to the thought-to-world forces that engender the grounds of a new or revised meaning. This requires creating the conditions that will lead to the right sort of effects at the thought level, which requires, among many other things, thinking about label-to-thought and world-to-thought causation. Thus, conceptual engineering projects that have goals as abstract as truth values still profit from paying attention to things as mundane and empirical as human psychology.

### Notion Engineering

The goal of conceptual engineering is to get certain changes in the mind or world to stick to achieve some purpose. Regardless of what the project is trying to make stick, conceptual engineers must focus on how labels, thoughts, and the world causally interact. This section argues that rather than avoiding this complexity, conceptual engineers should embrace it. I propose conceptual engineers talk about the combined package of label, thought, and worldly change as a *notion* and that conceptual engineers should design notions instead of standalone concepts, meanings, or words. This is because focusing just on labels, thoughts, or the world risks blinding conceptual engineers to the complexity of propagation.

Focusing merely on labels risks not accomplishing much of value. Labels are unpredictable and finicky; sometimes the inertia of language keeps problematic labels in circulation for centuries, while other times labels are dropped due to changes in slang. Moreover, labels will be interpreted differently by different people. Even when changing a label *is* useful, this usefulness comes from the label’s effects at the level of thought and the world. Dropping a hateful or misleading term may improve things, but it improves things because it leads to changes in inference patterns, behaviour, or abstracta. Therefore, labels can and should be paid attention to, but only as part of a larger package.

Focusing solely on changing thought risks either creating an unusable belief and/or (internalist) concept or setting oneself up to let an innovation moulder in the pages of academic journals. To pick an extreme example of creating an unusable concept by ignoring the world, imagine I engineered the conceptual content *planets closer to the Sun than Mercury*. There is certainly nothing stopping me from engineering such a concept. Such a planet is conceivable, metaphysically possible, and perhaps even epistemically possible. However, since there is no such planet, there is no use for such a concept outside of fictional or counterfactual uses, and people lack reason to adopt it (see Isaac, 2021). At the same time, if I came up with an extremely useful new concept but gave it a terrible label, people would struggle to adopt the concept in the same way philosophy students can struggle to remember the distinction between “de dicto” and “de re” statements or “pro tanto” vs “pro toto” reasons.

Focusing too much on thoughts or labels also risks unexpected results caused by the world. As Queloz and Bieber (2021) discuss, the preexisting examples we have of engineered or otherwise new concepts are full of unintended consequences, such as the repurposing of Nietzsche’s concepts “Übermensch” and “will to power” by antisemitic nationalists. Less drastically, recently the adjective *woke* shifted in just a few years from a term of praise among members of the political left to a derogatory term used by people on the right. Without a deep understanding of the environment in which we are introducing a label and/or mental state, we have little idea of how the term will actually be received. We also would not know if the concept introduces some sort of unintended injustice (Shields, 2021). How feasible those things are to predict is an open question (and likely best answered by sociologists and linguists), but any engineering project hoping to spread a certain behaviour, belief, mental representation, or label will be better placed to make key strategic decisions the more know about the environment in which it is being propagated.

Focusing too much on worldly changes, including altering abstracta or truth values, risks losing sight of whether a change is doable and how to go about that change. A possible word-meaning pair with certain truth conditions might be world-changing, but good consequences are not enough to propagate it. There are also the necessary questions of whether people can represent a token instantiation or ground of the necessary abstracta, whether people would want to have such a token instantiation or ground, and what would get people to form a corresponding token instantiation or ground. All three are questions of psychology, and as discussed in Sects. 3 and 4, the answer to the third question will involve the role of the label as a vehicle of the abstracta.

Designing a notion should be an empirical project. Because the relationships are complicated, causal, opaque from the armchair, and bear on the chances of success, they should be examined with the empirical tools of psychology, experimental linguistics, history, sociology, etc. Returning to labels as an illustration, while a well-designed label or name can carry information and help convey ideas, we need to understand people’s psychology to predict with any confidence that a given label will succeed at doing so. By their very nature, informativeness, transparency, and other label-to-thought causal forces depend on the mental representations of the individual encountering the label. A label that seems to carry information to one group of conceptual engineers may not read the same way to another group of conceptual engineers, let alone a normal person. Knowing if a label is a good label requires studying the people who we want to use the label, else we are just providing our idiosyncratic guesses. Because conceptual engineering needs to consider the complex web of causal forces affecting propagation that labels are merely one aspect of, and complex causal interactions are best studied empirically, conceptual engineering should be an empirical project.

## Conclusion

Conceptual engineering, regardless of the form it takes, is a complicated endeavour. Focusing too much on the target or goal of conceptual engineering risks making conceptual engineering look straightforward—even if the means to do so are not necessarily straightforward to identify or wield. In reality, conceptual engineers need to focus on a wide range of considerations and factors when trying to propagate what they want to propagate. To offer a way to conceptualise and understand the different interrelated causal factors at play in conceptual engineering, this paper suggested the (meta)framework of *labels, thoughts,* and *the world*. The label is the vehicle by which meaning, concepts, and ideas are presented. The thoughts are the token entities grounded in an individual’s psychological states. The world is all other stuff external to us. Because words are causally related to thoughts and thoughts are causally related to the world, any intervention at one level will affect and be affected by factors at the other levels. While attempts to understand these causal forces could be made from the armchair, doing so would unnecessarily hinder attempts at propagation. Instead, approaching these forces empirically would give conceptual engineers the best possible chance at successfully achieving their project’s purpose.

## Footnotes

1 The metaphysics is inspired by the semiotic triangle (Ogden and Richards 1930), although I do not intend to take on any substantive claims from Ogden and Richards.

2 Content externalists in the tradition of Burge (1979) may not like this use of “thought”. Unfortunately, I could not find a more neutral term.

3 As will become clear below, the labels of lexical items are not indirectly causally related to the grounds of every possible abstracta in theoretical space, e.g., platonist understandings of numbers. This paper nonetheless offers a way to think about how to engineer the labels of such abstracta to minimize unwanted mental representations. Thanks to Miguel Egler for this point.

4 *Slightly opaque* means the meaning of the adjective-noun phrase is not immediately clear from its parts. Imagine chefs discovered adding baking soda to butter makes the butter clear but otherwise unchanged. That butter would be clarified but it would not be clarified butter.

5 Good, useful, or trueness can come apart, and they will need to be weighed against each other according to the specifics of one’s framework. Thanks to Emilia Wilson for this point. Note that this is a newer formulation than in Landes (forthcoming).

6 On some conceptual engineering frameworks, the first question may be metaphysical, not empirical. The second question will always be empirical.

7 Beyond motivation and desiderata, abstracta relevant to conceptual engineering also play an indirect part in world-to-thought causation. While spooky entities such as Fregean concepts do not have a causal effect on our mental states, their grounds often do.
